# ﻿A new revision of the lichen genus *Pleopsidium* (Acarosporales, Acarosporaceae) in China reveals two new species

**DOI:** 10.3897/mycokeys.120.161566

**Published:** 2025-08-12

**Authors:** Min Ai, Cong Cong Miao, Fiona Ruth Worthy, Lazzat Nurtai, De Ning Zhang, Sheng Bang Zhang, Gui Lan Han, Li Song Wang, Xin Yu Wang

**Affiliations:** 1 State Key Laboratory of Phytochemistry and Natural Medicines, Kunming Institute of Botany, Chinese Academy of Sciences, Kunming, Yunnan 650201, China; 2 Yunnan Key Laboratory for Fungal Diversity and Green Development, Kunming Institute of Botany, Chinese Academy of Sciences, Kunming, Yunnan 650201, China; 3 Key Laboratory for Plant Diversity and Biogeography of East Asia, Kunming Institute of Botany, Chinese Academy of Sciences, Kunming, Yunnan 650201, China; 4 Key Laboratory of Plant Stress Research, College of Life Sciences, Shandong Normal University, Jinan, Shandong 250014, China; 5 Arid land Lichen Research Center of Western China, College of Life Science, Xinjiang University, Urumqi, Xinjiang 830046, China; 6 Qinghai Shanshui Natural Resources Survey Institute, Xining, Qinghai 810008, China; 7 Experimental Forest Farm of Datong Hui and Tu Autonomous County, Datong, Qinghai 810100, China

**Keywords:** Lichenized fungi, neotype, phylogeny, saxicolous yellow species, taxonomy

## Abstract

Based on a combination of morphological, chemical, and phylogenetic analyses, we present a new revision of the lichen genus *Pleopsidium* in China. *Pleopsidiumtumidulum***sp. nov.** and *P.corrugatulum***sp. nov.** are proposed as new species to science, both included in a distinctive clade (clade 1) together with *P.gobiense*. These three species are characterized by the absence of fatty acids, acaranoic and acarenoic acids, and share similar ecological traits. In contrast, species containing fatty acids, including *Pleopsidiumflavum*, *P.chlorophanum*, and *P.discurrens*, constitute a separate clade (clade 2). Fresh specimens of *Pleopsidiumdiscurrens* were collected from the type locality, Yulong Snow Mountain, and molecular data were generated to confirm the phylogenetic position of the species. Our study additionally reveals substantial morphological variation in this species. New collections and sequences of *Pleopsidiumgobiense* from Hami City, the type locality, are provided, from which a neotype is designated, confirming its phylogenetic position. Differences in morphology, anatomy, chemistry, and ecology among *Pleopsidium* species from China are discussed. A key and several phylograms are provided.

## ﻿Introduction

*Pleopsidium* Körb. was first described in 1855 (Körber 1855). In the early 20^th^ century, Magnusson (1924), Paulson (1925), and Räsänen (1943) considered *Pleopsidium* as a section of *Acarospora* A. Massal. After a detailed comparison with other similar species in *Acarospora* and *Lecanora* Ach., Hafellner (1993) reinstated *Pleopsidium* to the generic rank on the basis of ascus structure, pycnidia, upper cortex anatomy, secondary chemistry, habitat, ecology, and biogeography. Later phylogenetic research by Westberg et al. (2015) concluded that *Pleopsidium* species form a distinct group well separated from other genera in Acarosporaceae.

*Pleopsidium* includes four species characterized by a yellow upper surface, marginal lobes, *Pleopsidium*-type asci, and numerous spores. It also contains rhizocarpic acid and sometimes acaranoic, acarenoic, and lichesterinic fatty acids. Species of *Pleopsidium* are distributed in Antarctica, Asia, Europe, and North and South America (Wei 1991; Brodo et al. 2001; Nash III et al. 2007; Knudsen et al. 2008). *Pleopsidiumchlorophanum* (Wahlenb.) Arnold and *P.flavum* (Bellardi) Körb. were described from Europe (Hafellner 1993). Although they have been reported from China, these records need to be confirmed by molecular data (Paulson 1925, 1928; Moberg 1996; Niu et al. 2015, 2017; Aerguli et al. 2018). *Pleopsidiumgobiense* (H. Magn.) Hafellner is widely distributed in the deserts of Central Asia and has been reported from similar habitats in China (Magnusson 1940, 1944; Niu et al. 2015, 2017; Aerguli et al. 2018). *Pleopsidiumdiscurrens* (Zahlbr.) Obermayer was described from Yunnan and Sichuan and was later also reported from Tibet, China (Zahlbruckner 1930; Obermayer 1996).

The nomenclature was disputed for “*Pleopsidiumoxytonum* (Ach.) Rabenh.” and “*Pleopsidiumflavum* (Bell.) Körber,” but more recently, *Pleopsidiumoxytonum* has been treated as a later synonym of *P.flavum* (Knudsen and Arcadia 2018). *Pleopsidiumstenosporum* (Stizenb. ex Hasse) K. Knudsen, reported from North America, had also been treated as a synonym of *P.flavum* due to its similar morphology and chemistry (Knudsen 2011; Knudsen and Kocourková 2013). During our studies of *Pleopsidium* specimens in China, two new species were discovered based on integrated phenotypic and molecular analyses. These species are described and characterized below. In this study, we assign a total of six species to *Pleopsidium*.

## ﻿Materials and methods

### ﻿Morphological and chemical analyses

Specimens of *Pleopsidium* were mainly collected during expeditions across the Qinghai–Tibetan Plateau from 2018 to 2023. In this study, 130 specimens were examined and deposited in the Kunming Institute of Botany (China) herbarium (KUN) and the Arid Land Lichen Research Center of Western China, Xinjiang University (Urumqi, China) herbarium (XJU). Morphological and anatomical characters of thalli and apothecia were examined under a dissecting microscope (Nikon SMZ 745T) and an optical microscope (Nikon Eclipse Ci-S). Descriptions of the range of anatomical characters were based on the smallest and largest single values measured at 400× or 1000× magnification for all specimens. Photos were taken with a digital camera (Nikon Z5) and a stereomicroscope (Zeiss Axio Scope A1). The thallus and medulla were tested with K (a 10% aqueous solution of potassium hydroxide), C (a saturated aqueous solution of sodium hypochlorite), and P (a saturated solution of *p*-phenylenediamine in 95% ethyl alcohol) for identification. Secondary metabolites were identified using standardized thin-layer chromatography (TLC) with solvent system C (toluene:acetic acid = 85:15; Orange et al. 2001).

### ﻿DNA extraction, amplification, and sequencing

DNA was extracted from small fragments of fresh thallus tips or apothecia using a DNAsecure Plant Kit (TIANGEN) according to the manufacturer’s instructions. The following regions were amplified: the internal transcribed spacer region (ITS1–5.8S–ITS2), the nuclear large subunit rDNA gene (nuLSU), and the mitochondrial small subunit rDNA gene (mtSSU). The primers used were ITS1F (Gardes and Bruns 1993) and ITS4 (White et al. 1990), LR0R (Rehner and Samuels 1994) and LR5 (Vilgalys and Hester 1990), and mtSSU1 and mtSSU3R (Zoller et al. 1999), respectively. PCR amplifications were carried out in a 25 μL reaction volume containing 2 μL of genomic DNA, 1 μL of 10 mM solution for each primer, 12.5 μL of 2× T3 Taq PCR Mix (TSINGKE), and 8.5 μL of ddH_2_O. The PCR profile was initial denaturation at 94 °C for 5 min, followed by 30 cycles of 94 °C for 1 min, 54–56 °C for 1 min, and 72 °C for 1 min 30 s, with a final extension at 72 °C for 7 min in an automatic thermocycler. The PCR products were sequenced using the same amplification primers via Sanger technology by Sangon Biotech (Shanghai, China).

### ﻿Phylogenetic analyses

All raw sequences were assembled and edited using MAFFT v.7 in Geneious v8.0.2 with the default settings (Katoh et al. 2005; Katoh and Standley 2013). A 3-locus (nuITS–nuLSU–mtSSU) concatenated matrix was generated using *Pleopsidium* species sequences and sequences from related genera in Acarosporaceae (Table 1). Congruence between different gene regions was analyzed before combining sequences. The nuITS dataset consists of 57 newly generated sequences and 54 sequences from GenBank (Tables 1, 2). The nuLSU dataset consists of 21 sequences (Table 1). The final matrices are provided in the Suppl. materials 1, 2, 3: nuITS, nuLSU, and the 3-locus concatenated matrix. Bayesian inference (BI) and maximum likelihood (ML) were used to reconstruct the phylogenetic trees. Based on the lowest Bayesian information criterion (BIC), the best-fit partition substitution models were selected using PartitionFinder 2 (Lanfear et al. 2016) and RAxML (Stamatakis 2014) in PhyloSuite (Zhang et al. 2020) for the following ML and BI analyses: the nuITS dataset (TIM2e+G4) and the 3-locus concatenated matrix (GTR+I+G for each subset).

**Table 1.** Sequences and specimens in the phylogenetic tree. Newly obtained sequences are in bold. “Null” indicates that no sequences were available.

Taxon name	Voucher	Locality	GenBank accession number		
			nuITS	nuLSU	mtSSU
*Acarosporaschleicheri* 1	Obermayer 2919	China: Sichuan	LN810800	LN810800	LN810925
*A.schleicheri* 2	Sweat & Yansky KGS1196	USA: Arizona	LN810801	LN810801	LN810926
*A.umbilicata*	Tibell 23532 (UPS)	Sweden	LN810808	LN810808	LN810933
*Glypholeciaqinghaiensis* 1	22-71630	China	OP749916	NULL	OP749899
*G.qinghaiensis* 2	20-68255	China	MZ330789	NULL	OP749910
*G.scabra* 1	Westberg 08-232 (S)	Norway	LN810811	LN810811	LN810936
*G.scabra* 2	AFTOL-ID 1008	/	HQ650722	NULL	KJ766399
*G.scabra* 3	19-66159	China	MZ330790	NULL	OP749909
*Myriosporamyochroa*	Halda & Palice 1382	Czech	EU870677	NULL	EU870729
*M.rhagadiza*	Westberg 06-034	Sweden	EU870646	LN810876	EU870698
*M.scabrida*	Westberg 2824	Norway	EU870643	LN810877	EU870695
*M.smaragdula*	Wedin 6620 (UPS)	Sweden	EU870688	LN810879	EU870740
*M.tangerina*	Crewe 104 (UPS)	Greenland	EU870683	NULL	EU870735
*Pleopsidiumchlorophanum* 1	Nordin 6209 (UPS)	Sweden	LN810813	LN810813	LN810938
*P.chlorophanum* 2	Knudsen 16955	Austria	ON303961	ON303965	ON303852
*P.chlorophanum* 3	Nordin 4439 (UPS)	Sweden	EU870691	LN810881	EU870743
*P.chlorophanum* 4	Wedin 6589 (UPS)	Sweden	AY853384	AY853384	AY853335
*P.chlorophanum* 5	AFTOL-ID 1004 (DUKE)	/	NULL	DQ842017	DQ991756
*P.chlorophanum*	ZYY-137	Austria	PQ877185	PQ871181	PQ871158
*P.corrugatulum*	20151086-c	China: Ningxia	MN129010	MN129034	MN129047
*P.corrugatulum*	20130708-1	China: Xinjiang	MN129009	MN129033	MN129046
*P.corrugatulum*	ZSB23-906	China: Qinghai	PQ877160	PQ871169	PQ871146
*P.corrugatulum*	22-72992	China: Xinjiang	PQ877161	PQ871179	PQ871156
*P.corrugatulum*	22-72341	China: Xinjiang	PQ877162	PQ871180	PQ871157
*P.corrugatulum*	ZYJ1	China: Ningxia	PQ877163	PQ871182	PQ871159
*P.discurrens*	15-49590	China: Yunnan	MN129017	MN129040	MN129054
*P.discurrens*	17-56207	China: Yunnan	PQ877170	PQ871163	PQ871138
*P.discurrens*	17-55117a	China: Yunnan	PQ877171	PQ871164	PQ871139
*P.discurrens*	17-55054	China: Yunnan	PQ877172	PQ871165	PQ871140
*P.discurrens*	23-74275	China: Yunnan	PQ877175	NULL	PQ871143
*P.discurrens*	23-74395	China: Yunnan	PQ877176	NULL	PQ871144
*P.flavum* 1	Malicek	Czech	OK142757	OP497841	OK032142
*P.flavum* 2	Obermayer 7790	Austria	AY853385	AY853385	AY853336
*P.flavum* 3	Burgaz 2004 (H)	/	NULL	KJ766628	KJ766462
*P.flavum* 4	Spribille 40380 (GZU)	USA: Montana	NULL	NULL	MN508323
*P.gobiense* 1	AFTOL-ID 1003	/	HQ650723	DQ883698	DQ991755
*P.gobiense* 2	Moberg & Nordin (UPS)	Kazakhstan	DQ374144	NULL	DQ374123
*P.gobiense*	Pg161008-1	China: Xinjiang	MN129006	MN129030	MN129043
*P.gobiense*	Pc161016-1	China: Xinjiang	MN129007	MN129031	MN129044
*P.gobiense*	2014011A-1	China: Xinjiang	MN129008	MN129032	MN129045
*P.gobiense*	LD22-513	China: Xinjiang	PQ877166	PQ871177	PQ871154
*P.gobiense*	XY22-947	China: Xinjiang	PQ877167	PQ871178	PQ871155
*P.gobiense*	XJ-ZSB23-119	China: Xinjiang	PQ877165	PQ871173	PQ871150
*P.tumidulum*	18-58228	China: Qinghai	PQ877140	PQ871166	PQ871141
*P.tumidulum*	20157527-1	China: Neimenggu	PQ877141	PQ871167	PQ871142
*P.tumidulum*	ZSB24-1392	China: Qinghai	PQ877152	PQ871172	PQ871149
*P.tumidulum*	ZSB23-545	China: Qinghai	PQ877150	PQ871170	PQ871147
*P.tumidulum*	ZSB24-1363	China: Qinghai	PQ877147	PQ871168	PQ871145
*P.tumidulum*	ZSB24-1400	China: Qinghai	PQ877151	PQ871171	PQ871148
*P.tumidulum*	XY22-896	China: Xinjiang	PQ877155	PQ871175	PQ871152
*P.tumidulum*	ZYY22-558	China: Xinjiang	PQ877154	PQ871174	PQ871151
*P.tumidulum*	XY22-773	China: Xinjiang	PQ877156	PQ871176	PQ871153
*Polysporinasimplex*	Westberg SAR273 (S)	Austria	LN810826	LN810826	LN810951
*Po.subfuscescens*	Westberg 08-154 (S)	Norway	LN810832	LN810832	LN810958
*Pycnorasorophora*	Hermansson 7903a (UPS)	Sweden	FJ959357	AY853387	AY853338
*Sarcogynedistinguenda*	Westberg 08-305 (S)	Sweden	LN810854	LN810854	LN810979
*S.hypophaea*	Westberg SAR198 (S)	Sweden	LN810856	LN810856	LN810981
*S.regularis*	Westberg 08-034 (S)	Sweden	LN810861	LN810861	LN810986
*Timdaliaintricata* 1	Westberg P114 (S)	Sweden	LN810867	LN810867	LN810992
*T.intricata* 2	Westberg SAR92 (LD)	Sweden	LN810866	KP794969	LN810991

ML analyses were performed using IQ-TREE v.1.6.12 (Nguyen et al. 2015). Bootstrap frequencies were estimated from the consensus tree built with trees obtained from 2000 non-parametric bootstrapping pseudo-replicates. Bootstrap support values (MLBS) were obtained from the 70% majority rule tree of all saved trees. BI analyses were performed using MrBayes v3.2.6 (Ronquist et al. 2012). Markov chain Monte Carlo algorithms with four incrementally heated chains were run for 2 million generations to implement the BI analyses. Trees were sampled every 100 generations, and the first 25% of trees were discarded as burn-in. The stop rule was the average standard deviation of split frequencies < 0.01. Bayesian posterior probabilities (BPP) were obtained from the 95% majority rule consensus tree of all saved trees. The final trees were visualized using FigTree v1.4.0 (Rambaut 2012).

## ﻿Results

**Figure 1.**
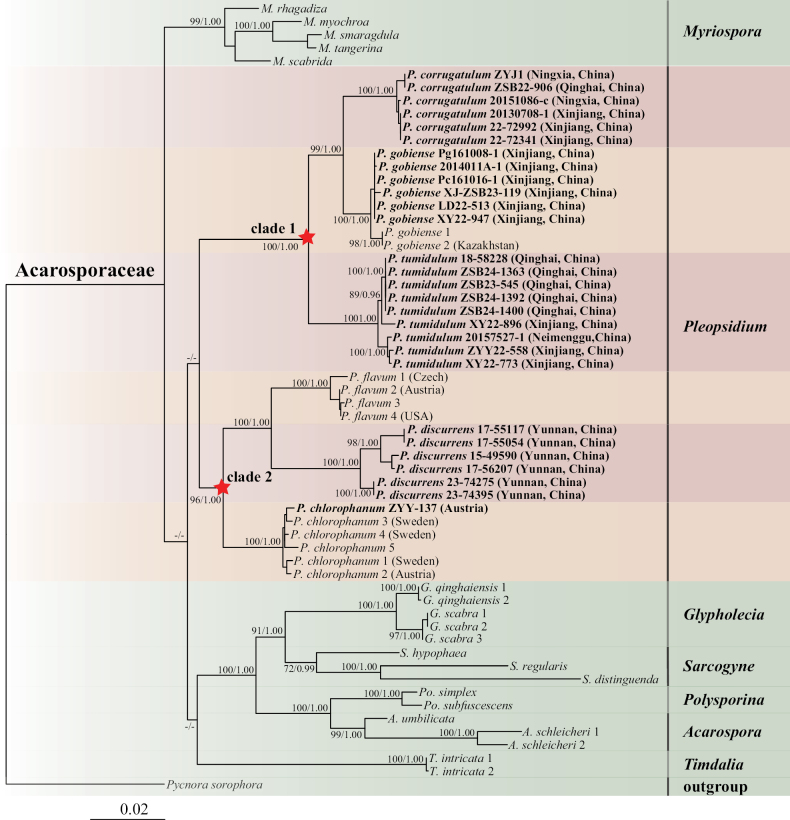
Maximum likelihood (ML) phylogenetic tree of *Pleopsidium* and selected Acarosporaceae species based on phylogenetic analysis of the nuITS–nuLSU–mtSSU concatenated sequence matrix. Newly obtained sequences are in bold. Maximum likelihood bootstrap support values (MLBS) and Bayesian posterior probabilities (PP) are given near the nodes.

The 3-locus (nuITS, nuLSU, and mtSSU) concatenated matrix comprised 167 terminals (513 bp for 57 nuITS, 838 bp for 50 nuLSU, and 751 bp for 60 mtSSU), which included 82 newly generated *Pleopsidium* sequences (Table 1). Six representative monophyletic genera—*Acarospora*, *Glypholecia* Nyl., *Myriospora* Nägeli ex Uloth, *Polysporina* Vězda, *Sarcogyne* Flot., and *Timdalia* Hafellner—were selected from Acarosporaceae, and *Pycnorasorophora* (Vain.) Hafellner was selected as the outgroup. The phylogenetic analyses showed that the genus *Pleopsidium* is located within Acarosporaceae, but this position was not highly supported (Fig. 1). Relationships between *Pleopsidium* and other genera within Acarosporaceae still require further research. Two distinct clades were formed within *Pleopsidium* (Fig. 1).

In clade 1, *Pleopsidiumgobiense*, *P.corrugatulum*, and *P.tumidulum* formed a monophyletic clade with strong support (100% BS and 1.00 PP). Specimens here described as *Pleopsidiumcorrugatulum* formed a well-supported sister clade to *P.gobiense* (100% BS and 1.00 PP). These two species differ in their anatomical characteristics. Therefore, we recognize *Pleopsidiumcorrugatulum* as a new species. The collections designated as *Pleopsidiumtumidulum* were clustered as a single lineage with high support (100% BS and 1.00 PP) and have also been introduced as a new species for China. These three species are similar in chemistry and ecology: they are characterized by the absence of fatty acids and share a Central Asian distribution. A detailed comparison of their differences is provided in the Discussion section.

In clade 2, *Pleopsidiumflavum*, *P.chlorophanum*, and *P.discurrens* formed an independent clade with high support (96% BS and 1.00 PP). *Pleopsidiumflavum* (the type species of *Pleopsidium*) formed a single clade with strong support (100% ML and 1.00 PP). *Pleopsidiumchlorophanum* and *P.discurrens* also formed a distinct monophyletic clade with high support (100% ML and 1.00 PP). Fatty acids were detected within the thalli of all three species (Hafellner 1993; Obermayer 1996). Sequences of *Pleopsidiumflavum* and *P.chlorophanum* were downloaded from GenBank. Specimen No. ZYY-137 is preserved at the KUN herbarium but was originally collected from Austria. The distribution of these two species has not previously been confirmed in China. A detailed discussion is given below.

## ﻿Discussion

### ﻿Phylogeny of *Pleopsidium*

**Figure 2.**
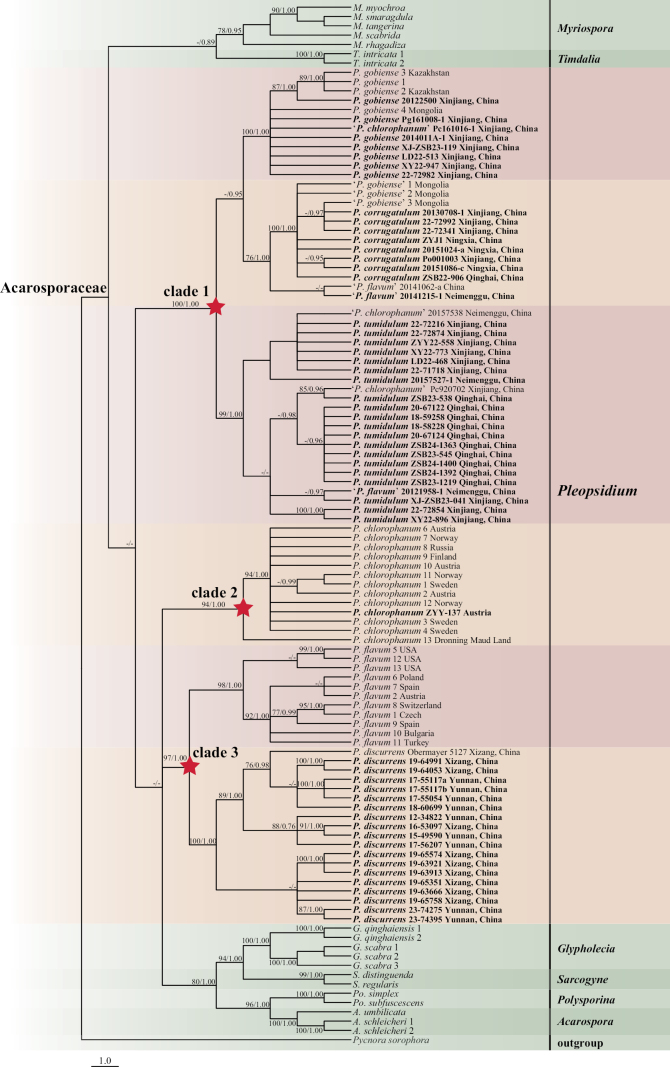
Maximum likelihood (ML) phylogenetic tree of the genus *Pleopsidium* and selected species of Acarosporaceae based on phylogenetic analysis of the nuITS sequence matrix. Newly obtained sequences are in bold. Maximum likelihood bootstrap support values (MLBS) and Bayesian posterior probabilities (PP) are given near the nodes.

The phylogenetic trees illustrate that *Pleopsidium* species form two highly supported monophyletic lineages (Fig. 1), both in the single-region phylogeny (nuITS; Fig. 2) and the combined-region phylogeny (nuITS–nuLSU–mtSSU; Fig. 1). Species within clade 1 do not contain fatty acids, whereas species within clade 2 contain fatty acids such as acaranoic or acarenoic acids. These two clades could be treated as different genera based on chemical and molecular evidence. The genus concept of *Pleopsidium* would then require revision, with species that lack fatty acids being excluded from *Pleopsidium*. However, such a taxonomic proposal would be unsupported by morphological evidence. The relationships between these two clades and other Acarosporaceae genera require further clarification. We therefore maintain the current circumscription of the genus. The phylogeny of Acarosporaceae requires further research, including the collection of additional samples from other regions of the world and molecular data for other genera within Acarosporaceae.

**Table 2.** nuITS sequences and specimens. Newly obtained sequences are in bold.

Taxon name	Voucher	Locality	nuITS GenBank accession number
*Pleopsidiumchlorophanum* 6	Keller 15669	Austria	DQ525482
*P.chlorophanum* 7	Haugan SK00-117	Norway	DQ525484
*P.chlorophanum* 8	Nimis & Tretiach 25181 (TSB)	Russia	DQ525488
*P.chlorophanum* 9	Sipman Tan & Reiniko 23368	Finland	DQ525489
*P.chlorophanum* 10	Arup L98077	Austria	EF535222
*P.chlorophanum* 11	O-L-184414	Norway	MK812213
*P.chlorophanum* 12	O-L-208244	Norway	MK812354
*P.chlorophanum* 13	Engelskjøn (BGL-500713)	Dronning Maud Land	DQ525485
*P.corrugatulum*	20151024-a	China: Ningxia	PQ877158
*P.corrugatulum*	Po001003	China: Xinjiang	MN129011
‘*P.flavum*’	20141215-1	China: Neimenggu	PQ877159
‘*P.flavum*’	20141062-a	China	MF188905
‘*P.gobiense*’ 1	Huneck MVR88-47 (B600095250)	Mongolia	DQ525492
‘*P.gobiense*’ 2	Huneck MVR88-304 (B600095255)	Mongolia	DQ525493
‘*P.gobiense*’ 3	Reeb VR 14-VII-04/6 (DUKE)	Mongolia	DQ525494
*P.discurrens*	Obermayer 5127 (GZU)	China: Xizang	DQ525510
*P.discurrens*	16-53097	China: Xizang	PQ877169
*P.discurrens*	17-55117b	China: Yunnan	MN129016
*P.discurrens*	12-34822	China: Yunnan	PQ877173
*P.discurrens*	18-60699	China: Yunnan	PQ877174
*P.discurrens*	19-65574	China: Xizang	PQ877177
*P.discurrens*	19-63921	China: Xizang	PQ877179
*P.discurrens*	19-64991	China: Xizang	PQ877180
*P.discurrens*	19-64053	China: Xizang	PQ877181
*P.discurrens*	19-63666	China: Xizang	PQ877182
*P.discurrens*	19-63913	China: Xizang	PQ877183
*P.discurrens*	19-65758	China: Xizang	PQ877184
*P.discurrens*	19-65351	China: Xizang	PQ877178
*P.flavum* 5	M0062614	USA	DQ525500
*P.flavum* 6	Reeb VR 22-VIII-99/4b (DUKE)	Poland	DQ525507
*P.flavum* 7	Reeb VR 2-IX-00/15 (DUKE)	Spain	DQ525521
*P.flavum* 8	Grisons, Scheidegger	Switzerland	DQ525514
*P.flavum* 9	Reeb VR 31-VIII-00/1 (DUKE)	Spain	DQ525515
*P.flavum* 10	B600125577	Bulgaria	DQ525516
*P.flavum* 11	John & Zeybek 6.167 (B600125572)	Turkey	DQ525517
*P.flavum* 12	Leavitt 18-300 BRY-C	USA: Utah	MZ243480
*P.flavum* 13	Leavitt 18-355 BRY-C	USA: Utah	MZ243479
*P.gobiense* 3	Vost, Moberg & Nordin (M0062472)	Kazakhstan	DQ525496
*P.gobiense* 4	Golubkova & Zogt 431 (DUKE)	Mongolia	DQ525497
*P.gobiense*	20122500	China: Xinjiang	PQ877164
*P.gobiense*	22-72982	China: Xinjiang	PQ877168
*P.tumidulum*	18-59258	China: Qinghai	PQ877139
*P.tumidulum*	20-67122	China: Qinghai	PQ882529
*P.tumidulum*	22-71718	China: Xinjiang	PQ882528
*P.tumidulum*	20-67124	China: Qinghai	PQ877143
*P.tumidulum*	22-72854	China: Xinjiang	PQ877145
*P.tumidulum*	ZSB23-1219	China: Qinghai	PQ877153
*P.tumidulum*	XJ-ZSB23-041	China: Xinjiang	PQ877148
*P.tumidulum*	ZSB23-538	China: Qinghai	PQ877149
*P.tumidulum*	LD22-468	China: Xinjiang	PQ877157
*P.tumidulum*	22-72874	China: Xinjiang	PQ877146
*P.tumidulum*	22-72216	China: Xinjiang	PQ877144
‘*P.flavum*’	20121958-1	China: Neimenggu	PQ877142
‘*P.chlorophanum*’	20157538	China: Neimenggu	MF188904
‘*P.chlorophanum*’	Pc920702	China: Xinjiang	MF188906

### ﻿Species delimitation of *Pleopsidium*

Species in *Pleopsidium* have similar morphological and chemical characters. Molecular data could help to resolve and identify such cryptic taxa.

The three species in clade 1 (Fig. 1) share the absence of fatty acids but show morphological differences. *Pleopsidiumtumidulum* has convex, plump, swollen, and smooth long lobes (2.5–4.5 mm), a thick thallus (up to 1.5–3.0 mm), and can be reliably recognized by its wide apothecia (1.5–2.5 mm). Both *Pleopsidiumcorrugatulum* and *P.gobiense* have thinner thalli (<1.5 mm), short lobes (<3 mm long), and smaller apothecia (up to 1.25 mm). There are only minor morphological differences between *Pleopsidiumcorrugatulum* and *P.gobiense*, potentially due to their similar habitats. *Pleopsidiumgobiense* has numerous apothecia and few pycnidia, and the apothecia are plane, nearly flat to the thallus. In *Pleopsidiumcorrugatulum*, only a few specimens have apothecia, whereas most have many pycnidia.

**Figure 3.**
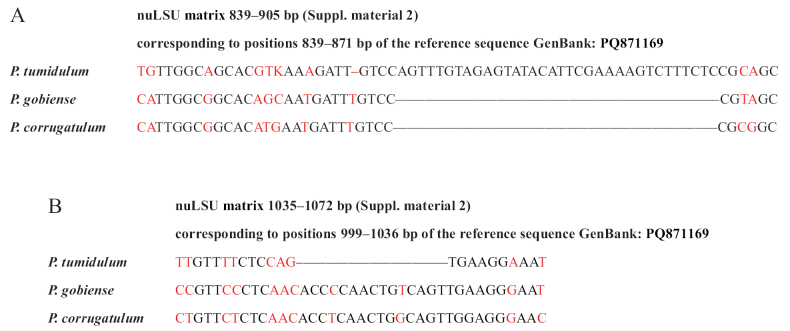
Alignment of nucleotide sequences within a portion of nuLSU, allowing identification of the three recognized species that lack fatty acids. Deviations are in red font, and short lines represent nucleotides that are not present. The IUPAC ambiguity code is used. A. Position 839–905 bp; B. Position 1035–1072 bp.

Molecular data provided significant support for distinguishing among these three species. The nuLSU gene was highly variable (Fig. 3). For nuLSU barcode sequences designed to identify these three species, BLASTn top-scoring hits matched the species identity inferred from our phylogenetic inferences (Suppl. material 4), demonstrating the reliability of the short barcodes.

The specimens of *Pleopsidiumdiscurrens* in clade 2 (Fig. 1) showed variable morphology, but few molecular differences were detected. Materials from the type locality, the Hengduan Mountains region, showed typical ribbon-like, elongate, branched, and spreading lobes, which is the distinctive character of this species. However, specimens from alpine desert regions had different morphology, with much shorter and more aggregated lobes. Molecular data indicate that they belong to the same species (Fig. 2). The morphological differences may be due to adaptation to a drier environment. This warrants further investigation using specimens collected across different climatic zones.

### ﻿Misidentifications

*Pleopsidiumchlorophanum* was reported by Paulson (1925, 1928) and referenced also by Zahlbruckner (1930) and Obermayer (1996) from Mount Everest and northwestern Yunnan in China. We examined specimens from Yunnan, Xizang, Xinjiang, Qinghai, Ningxia, and Neimenggu provinces in China but did not find further examples of the species. Niu et al. (2017) also reported *Pleopsidiumchlorophanum* but detected calycin in those specimens—a compound known to be present in morphologically similar species of *Lecanora* (Yakovchenko et al. 2019). We re-examined specimens [02-21440 (KUN), 04-25611 (KUN), and 14-45562 (KUN)] that had eight-spored asci and confirmed that they clearly did not belong to *Pleopsidium* but were misidentifications of *Lecanora*. Aerguli et al. (2018) identified some specimens from Xinjiang and Neimenggu as *Pleopsidiumchlorophanum*. Sequences of the two specimens (20157538, Pc920702) cited by Aerguli et al. (2018) were clustered with *Pleopsidiumtumidulum*. These two specimens both lacked fatty acids. The specimen Pc161016-1 also lacked fatty acids, and its sequence clustered with *Pleopsidiumgobiense* (Fig. 2).

Some old specimens from Xizang and Yunnan had been identified as *Pleopsidiumflavum* by Niu et al. (2017). However, we have surveyed those regions on multiple occasions and only found samples of *Pleopsidiumdiscurrens*. Some specimens with atypical morphology of *Pleopsidiumdiscurrens* could easily be misidentified as *P.flavum*. After examining additional specimens, we re-identified five specimens cited as *Pleopsidiumflavum* by Niu et al. (2017) as *P.discurrens*. Four specimens classified as *Pleopsidiumflavum* by Aerguli et al. (2018) were also revised based on chemical and molecular analyses: *P.corrugatulum* (20151068, 20141215-1, 20141062-a) and *P.tumidulum* (20121958-1) (Fig. 2).

Despite extensive field surveys and phylogenetic analysis, we have not found *Pleopsidiumchlorophanum* or *P.flavum* in China. Further comprehensive surveys are required to confirm whether these species are present in the country.

### ﻿Taxonomy

#### ﻿ Pleopsidium corrugatulum

Taxon classificationFungiAcarosporalesAcarosporaceae

C. C. Miao, Xin Y. Wang & Li S. Wang sp. nov.

93F151F7-7968-5DDD-89E7-678AD0307C2D

859408

Figs 4 , 5

##### Type.

China. • Qinghai Prov.: Xining Ci., 36°33'17"N, 101°54'01"E, alt. 2190 m, 07 Jul. 2023, S.B. Zhang et al. ZSB23-906 (holotype: KUN; isotype: SDNU).

##### Diagnosis.

The new species differs from *Pleopsidiumtumidulum* by its wrinkled areoles surface, thinner thalli, shorter lobes, numerous pycnidia, and molecular data.

##### Description.

Thallus crustose, tightly attached to the substrate, areolate at center, radiate-plicate at margin; areoles rimose, wrinkled, mostly infertile, 1–2.3 × 0.8–1.5 mm; marginal lobes sometimes cracked, 1–3 mm long, 1–2 mm wide, 0.7–1.5 mm thick, tightly attached to the substrate; upper surface yellow or dark yellowish green; upper cortex yellow, 25–50 μm thick; algal layer 100–175 μm thick; medulla white, 100–275 μm thick. Apothecia rare to moderately frequent, one mature apothecium or several young ones in each areola, cryptolecanorine to lecanorine, roundish, 0.9–1.25 mm wide; disc pale yellow, darker than thallus, plane; margin yellow, concolorous with thallus; epihymenium brownish yellow, 25–50 μm thick; hymenium hyaline, 37.5–62.5 μm high; paraphyses simple, with 4–5 septa, apices often expanded; hypothecium hyaline, 50–87.5 μm high; asci clavate, 37.5–55 × 12.5–20 μm, ±100-spored; ascospores hyaline, ellipsoidal, aseptate, 5–6 × 2–3 μm. Pycnidia immersed; conidia bacilliform, 2–2.5 × 1 μm.

**Figure 4.**
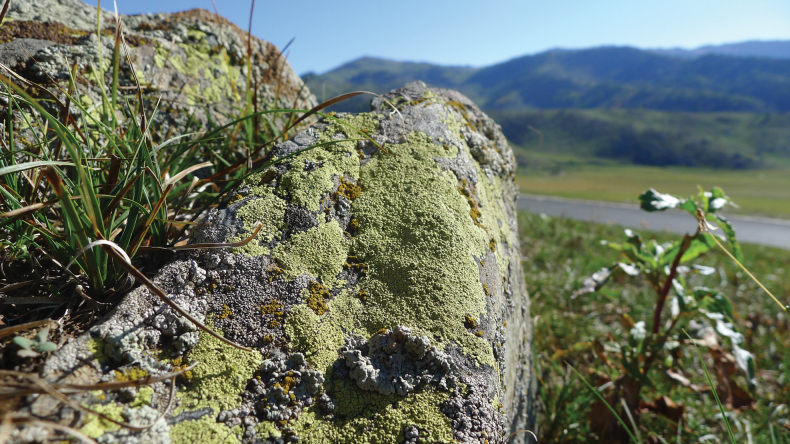
*Pleopsidiumcorrugatulum* in its habitat.

**Figure 5.**
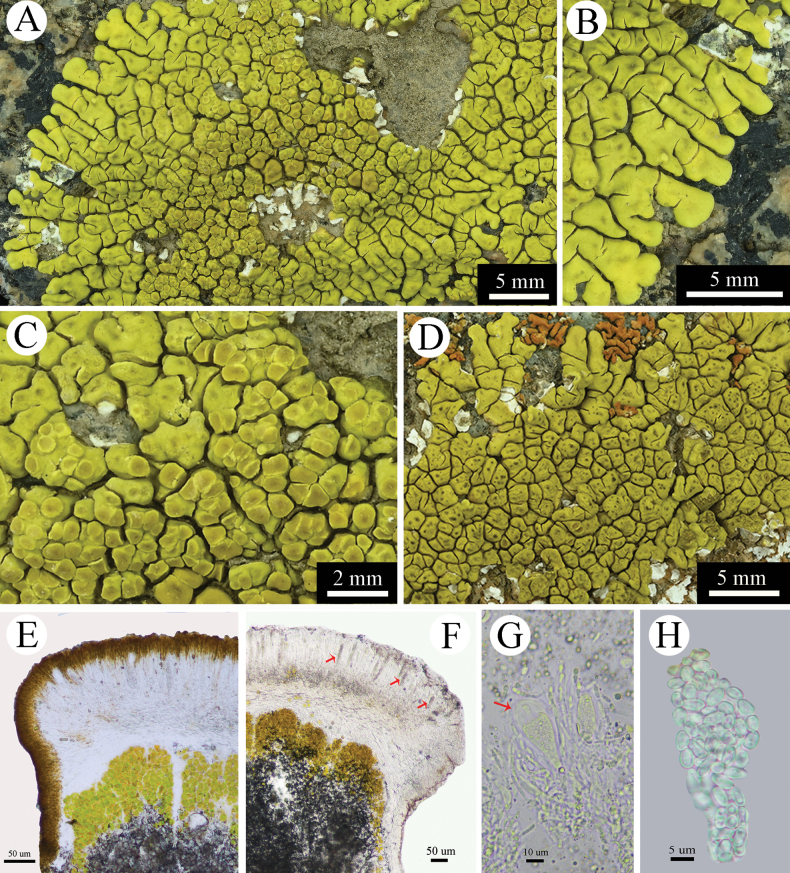
*Pleopsidiumcorrugatulum* (ZSB23-906 KUN). A. Thallus; B. Lobes; C. Apothecia; D. Pycnidia; E, F. Section of apothecium; G. Ascus containing ascospores; H. Simple ascospores. Scale bars: 5 µm (H); 10 µm (G); 50 µm (E, F); 2 mm (C); 5 mm (A, B, D).

##### Chemistry.

Thallus UV+ dark orange-yellow, P–, K–, C–, KC–. Containing rhizocarpic acid.

##### Distribution and ecology.

This species usually grows on exposed rocks in the Gobi, desert steppe, and in bush formations between ca 1290 m and ca 2190 m. It is distributed in the Qinghai, Xinjiang, and Ningxia provinces in China.

##### Etymology.

The epithet “*corrugatulum*” refers to the wrinkled surface of the species.

##### Note.

It is characterized by the absence of apothecia in most examined specimens, wrinkled surface, rugose lobes, numerous pycnidia, and absence of fatty acids. *Pleopsidiumgobiense* also lacks fatty acids but frequently develops apothecia with flat surfaces.

##### Selected specimens examined.

China. • **Ningxia Prov.**: Yinchuan Ci., Gunzhongkou Scenic Spot, 26 Jul. 2021, Y.J. Zhang ZYJ1 (KUN); • Helan Mount Nature Reserve, Suyukou Forest Park, 38°44'13"N, 105°57'10"E, alt. 1640 m, 19 Aug. 2015, Abdulla Abbas 20151024-a (XJU); • Helan Mount Nature Reserve, 38°44'06"N, 105°57'10"E, alt. 1650 m, 13 Aug. 2015, Abdulla Abbas 20151086-c (XJU). • **Xinjiang Prov.**: Hami Ci., Balikun Co., G335, 43°36'09"N, 92°44'45"E, alt. 1639 m, 04 Jul. 2022, L.S. Wang et al. 22-72992 (KUN); • Hami Ci., Balikun Co., Sa’erqiaoke Vil., 43°45'57"N, 92°02'26"E, 04 Jul. 2022, L.S. Wang et al. 22-72341 (KUN); • Fuhai Co., Hongshanzui, alt. 2000 m, 3 Oct. 2000, Abdulla Abbas Po001003 (XJU); • Yili Region, Altay Qinggou, alt. 1294 m, 17 June 2017, Abdulla Abbas 20130708-1 (XJU).

#### ﻿ Pleopsidium tumidulum

Taxon classificationFungiAcarosporalesAcarosporaceae

M. Ai, Xin Y. Wang & Li S. Wang sp. nov.

10CA080F-4856-572D-AA3C-FA91392E20DD

859409

Figs 6 , 7

##### Type.

China. • Qinghai Prov.: Dulan Co., Xiangjia Vil., 36°12'19"N, 97°01'00"E, alt. ca 2850 m, on rock, 15 Sep. 2020, L.S. Wang et al. 20-67124 (holotype: KUN; isotype: SDNU).

##### Diagnosis.

The new species differs from other *Pleopsidium* species by lacking fatty acids, having thick thalli, convex, plump, and swollen lobes, and by molecular data.

##### Description.

Thallus crustose, tightly attached to the substrate, areolate at center, radiate-plicate at margins; areoles approximately uniform, closely linked, fertile, 1.5–2.5 × 1.0–1.8 mm; marginal lobes long, convex, and plump, 2.5–4.5 mm long, 0.8–2.5 mm wide, 1.5–3.0 mm thick, tightly attached to the substrate; the apices of marginal lobes have lower cortex; upper surface bright yellow with rhizocarpic acid in cortex, smooth, becoming convex when mature; upper cortex yellow, 25–37.5 μm thick; algal layer 62.5–100 μm thick; medulla white, more than 87.5 μm thick; areoles at the center without the lower cortex. Apothecia numerous, one apothecia in each areola, cryptolecanorine to lecanorine, roundish, 1.3–2.5 mm in diam.; disc yellow, darker than thallus, plane to slight convex; margins yellow, concolorous with thallus; epihymenium brownish yellow, 25–37.5 μm thick; hymenium hyaline, 100–112.5 μm high; paraphyses simple, with 4–5 septa, apices often expanded; hypothecium hyaline, 50–62.5 μm high; asci clavate, 37.5–55 × 17.5–25 μm, ±100-spored; ascospores hyaline, ellipsoidal, aseptate, 4–5 × 2–3 μm. Pycnidia immersed; conidia bacilliform, 2–3 × 1 μm.

**Figure 6.**
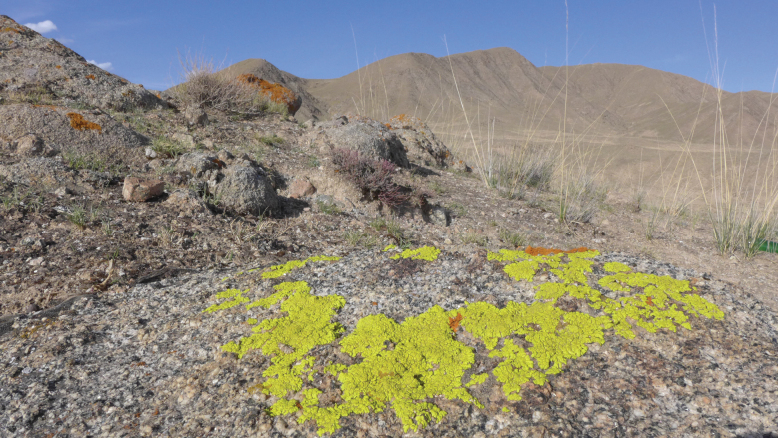
*Pleopsidiumtumidulum* in its habitat (18-59258 KUN).

**Figure 7.**
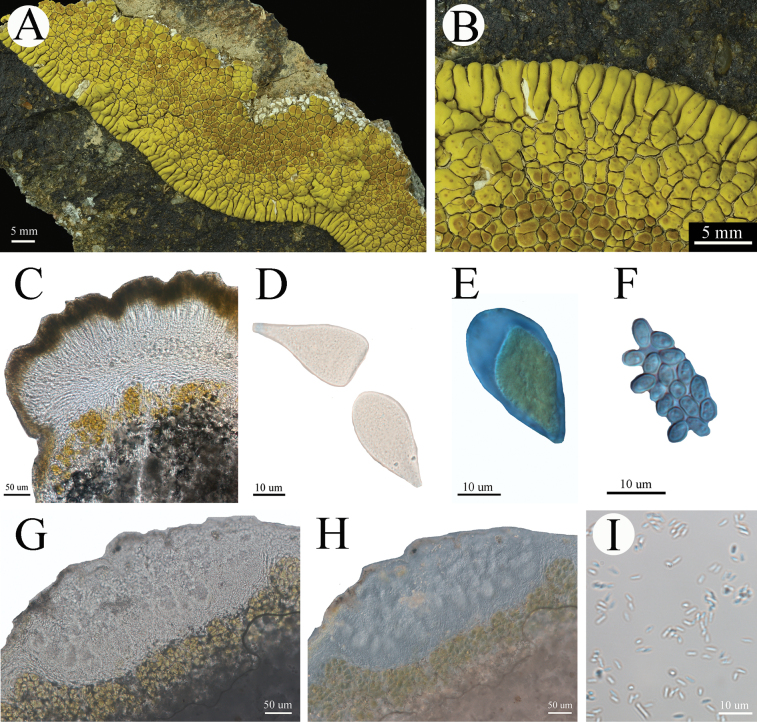
*Pleopsidiumtumidulum* (20-67124 KUN). A. Thallus; B. Lobes and apothecia; C. Section of apothecium; D, E. Young asci; F. Simple ascospores; G, H. Section of pycnidia; I. Conidia. E, F, H were captured under polarized light. Scale bars: 10 µm (D, E, F, I); 50 µm (C, G, H); 5 mm (A, B).

##### Chemistry.

Thallus UV+ dark orange-yellow, P–, K–, C–, KC–. Containing rhizocarpic acid and an unknown acid (trace).

##### Distribution and ecology.

This species usually grows on exposed rocks in the Gobi, desert steppe, and in bush formations at elevations between ca 1540 m and ca 3470 m. It is distributed in the Qinghai, Xinjiang, and Neimenggu provinces in China.

##### Etymology.

The epithet “*tumidulum*” refers to its plump and swollen lobes.

##### Note.

It is morphologically similar to *Pleopsidiumgobiense* and *P.corrugatulum*, but *P.tumidulum* has thicker and smoother thalli (up to 3 mm thick), longer marginal lobes (up to 4.5 mm long), bigger apothecia (over 1.5 mm), and smaller ascospores (less than 5 μm long). The new species differs from clade 2 *Pleopsidium* species (*P.discurrens*, *P.chlorophanum*, and *P.flavum*) in lacking fatty acids.

##### Selected specimens examined.

China. • **Neimenggu Prov.**: Huhehaote Ci., Luotuogou Vil., 42°56'22"N, 86°09'05"E, alt. 2680 m, 11 Aug. 2012, Lazzat Nurtai 20121958-1 (XJU); • Alxa Youqi, 39°04'27"N, 101°01'10"E, alt. 2307 m, 17 Aug. 2015, Lazzat Nurtai 20157527-1 (XJU). • **Qinghai Prov.**: Wulan Co., Gobi desert along the way from Chaka to Wulan, 36°52'18"N, 98°55'45"E, alt. 3191 m,19 May 2018, L.S. Wang et al. 18-58228 (KUN); • Wulan Co., Gobi desert along the way from Chaka to Wulan, 36°52'18"N, 98°55'45"E, alt. 3151 m, 19 May 2018, L.S. Wang et al. 18-59258 (KUN); • Wulan Co., Dulan Lake, 36°48'34"N, 98°27'06"E, alt. 2905 m, 25 Mar. 2023, S.B. Zhang et al. ZSB23-545 (KUN); • Wulan Co., Halihatu Forest Park, 37°00'41"N, 98°40'27"E, alt. 3215 m, 24 Mar. 2023, S.B. Zhang et al. ZSB23-538 (KUN); • Dulan Co., Kaoxiaotu Scenic Spot, 36°07'03"N, 98°06'23"E, alt. 3399 m, 20 Apr. 2024, S.B. Zhang et al. ZSB24-1392 (KUN); • Dulan Co., Reshui Vil., 36°13'02"N, 98°09'07"E, alt. 3245 m, 20 Apr. 2024, S.B. Zhang et al. ZSB24-1400 (KUN); • Dulan Co., Xiangjia Vil., 36°12'19"N, 97°01'00"E, alt. 2856 m, 15 Sep. 2020, L.S. Wang et al. 20-67122 (KUN); • Datong Co. Xiegou Vil., 36°55'42"N, 101°32'12"E, alt. 3090 m, 04 Mar. 2025, S.B. Zhang et al. ZSB25-2149 (KUN); • Dachaidan Administrative District, Snow Mountain Hot Spring Tourist Resort, 37°56'00"N, 95°22'54"E, alt. 2593 m, 16 Sep. 2023, S.B. Zhang et al. ZSB23-1219 (KUN); • Delingha Ci, Cypress Mountain, 37°28'34"N, 97°21'37"E, Alt. 3469 m, 17 Apr. 2024, S.B. Zhang et al. ZSB24-1363 (KUN). • **Xinjiang Prov.**: Hami Ci., Balikun Co., Haiziyan Vil., 43°35'49"N, 92°46'28"E, alt. 1543 m, 05 Sep. 2023, S.B. Zhang et al. XJ-ZSB23-041 (KUN); • Hejing Co., G218, 42°59'40"N, 86°06'22"E, alt. 3003 m, 01 Jul. 2022, L.S. Wang et al. 22-72854 (KUN); • Hejing Co., G218, 42°54'22"N, 86°17'37"E, alt. 2221 m, 01 Jul. 2022, L.S. Wang et al. 22-72874 (KUN); • Hejing Co., G218, 42°54'23"N, 86°17'39"E, alt. 2234 m, 01 Jul. 2022, D. Liu LD22-468 (KUN); • Hejing Co., Tianshan Mountain, 42°50'40"N, 86°21'05"E, alt. 1951 m, 29 Jun. 2022, Y.Y. Zhang ZYY22-558 (KUN), L.S. Wang et al. 22-71718 (KUN); • Hejing Co., Baluntai Town, 42°51'50"N, 86°26'35"E, alt. 2036 m, 02 Jul. 2022, X.Y. Wang et al. XY22-896 (KUN); • Hejing Co., Baluntai Town, 42°50'40"N, 86°21'06"E, alt. 1904 m, 29 Jun. 2022, X.Y. Wang et al. XY22-773 (KUN), L.S. Wang et al. 22-72216 (KUN).

#### ﻿ Pleopsidium discurrens

Taxon classificationFungiAcarosporalesAcarosporaceae

(Zahlbr.) Obermayer, Annales Botanici Fennici 33(3): 232 (1996)

9A9F7FD8-20A0-51D7-88C6-1D024574F273

Fig. 8

≡ Acarospora (sect. Pleopsidium) discurrens A. Zahlbr. in Handel-Mazzetti, Symbolae Sinicae, Pars III:140-141, 1930.^1^

##### Ind Loc.

“China. Prov. Yünnan [=Yunnan] bor.-occid.: In montis Yülung-schan prope urbem Lidjiang (“Likiang”) regione alpina, ad rupes inter pratum Latuka et alveum Schitako. Substr. schisto argilloso, alt. s. m. ca. 4000 m, leg. 20. Vll. 1914, Dr. Heinr. Frh. v. Handel-Mazzeni. (Diar. Nr. 676). det. Zahlbruckner Nr. 4297. (lectotype: WU; isolectotype: W)”.

##### Note.

**Figure 8.**
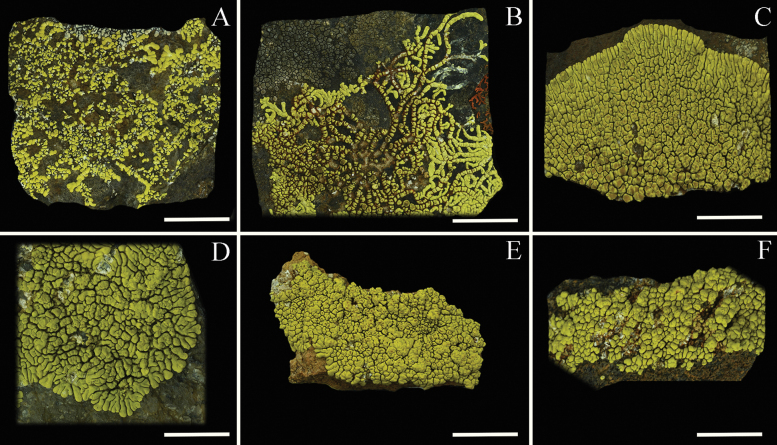
Intraspecific morphological variation of *Pleopsidiumdiscurrens*. A. 15-49590 (KUN), Yunnan; B. 17-56207 (KUN), Yunnan; C 17-55054 (KUN), Yunnan; D. 19-65758 (KUN), Xizang; E. 19-64991 (KUN), Xizang; F. 19-63913 (KUN), Xizang. Scale bars: 5 mm.

*Pleopsidiumdiscurrens* was described from Yulong Snow Mountain of Yunnan and Sichuan and then later also found in Xizang (Zahlbruckner 1930; Obermayer 1996). It was defined as having typical ribbon-like, elongate, branched, and spreading lobes (Fig. 8B). However, in combination with molecular systematics, this species shows extensive intraspecific morphological variation (Fig. 8). The specimens from northwestern Yunnan often have typical lobes (Fig. 8A, B), while lobes of specimens from Xizang are non-branched (Fig. 8E, F).

##### Selected specimens examined.

China. • **Yunnan Prov.**: Deqing Co., Baima Snow Mountain, 28°23'17"N, 99°01'10"E, alt. 4400 m, 01 Nov. 2015, L.S. Wang et al. 15-49590 (KUN); • Deqing Co., Baima Snow Mountain, 28°23'17"N, 99°01'02"E, alt. 4519 m, 4 Jul. 2012, L.S. Wang et al. 12-34822 (KUN); • Lijiang Ci., Yulong Naxi Autonomous Co., Yulong Snow Mountain, 27°01'56"N, 100°11'20"E, alt. 3737 m, 24 Oct. 2017, A.C. Yin 17-56207 (KUN); • Lijiang Ci., Laojunshan Mountain, 26°38'30"N, 99°43'47"E, alt. 3855 m, 28 Sep. 2018, L.S. Wang et al. 18-60699 (KUN); • Shangri-la Ci., on the way from Shangri-La to Daocheng, 28°26'00"N, 100°13'38"E, alt. 2951 m, 2 Jun. 2023, L.S. Wang et al. 23-74275 (KUN); • Shangri-la Ci., on the way from Shangri-La to Daocheng, 28°26'00"N, 100°13'39"E, alt. 2981 m, 2 Jun. 2023, L.S. Wang et al. 23-74395 (KUN); • Kunming Ci., Dongchuan District, Guniuzhai Mountain Scenic Spot, 26°10'01"N, 103°13'33"E, alt. 3190 m, 10 May. 2017, L.S. Wang et al. 17-55117 (KUN); • Kunming Ci., Dongchuan District, Guniuzhai Mountain Scenic Spot, 26°09'50"N, 103°13'31"E, alt. 3100 m, 10 May. 2017, L.S. Wang et al. 17-55054 (KUN). • **Xizang Prov.**: Zuogong Co., on the way from Rumei to Zuogong, at the 3550 milestone on G318, 29°43'16"N, 98°01'34"E, alt. 4968 m, 21 Sep. 2016, L.S. Wang et al. 16-53097 (KUN); • Dingri Co., Zhaguo Vil., 28°35'25"N, 86°53'58"E, alt. 4298 m, 27 Jul. 2019, L.S. Wang et al. 19-65574 (KUN); • Nielamu Co., Yalai Vil., 28°21'52"N, 86°05'44"E, alt. 4402 m, 26 Jul. 2019, L.S. Wang et al. 19-64991 (KUN); • Nielamu Co., 28°21'51"N, 86°05'42"E, alt. 4382 m, 26 Jul. 2019, L.S. Wang et al. 19-64053 (KUN); • Cuoqin Co., S206, 31°03'34"N, 85°02'11"E, alt. 4839 m, 20 Jul. 2019, L.S. Wang et al. 19-63666 (KUN); • Pulan Co., La’angcuo Lake, 30°34'50"N, 81°15'25"E, alt. 4628 m, 24 Jul. 2019, L.S. Wang et al. 19-63913 (KUN), L.S. Wang et al. 19-63921 (KUN); • Dingjie Co., Chentang Town, 27°55'48"N, 87°37'57"E, alt. 4243 m, 28 Jul. 2019, L.S. Wang et al. 19-65758 (KUN).

#### ﻿ Pleopsidium gobiense

Taxon classificationFungiAcarosporalesAcarosporaceae

(H. Magn.) Hafellner, Nova Hedwigia 56(3–4): 294 (1993)

193F4997-096B-55F2-8751-C46FB2DA401B

MBT 10027662

Fig. 9

≡ Acarosporagobiensis H. Magn., K. Svenska Vetensk-Akad. Handl., Ser. III 7(4): 98, 1929.

##### Ind Loc.

“Gobi Wüste, Lage VII SO von Chami 1895 Futterer”.

##### Type.

• China. Xinjiang Prov.: Hami Ci., Balikun Co., Haiziyan Vil., 43°36'10"N, 92°44'43"E, alt. ca 1570 m, on rock, 04 Jul. 2022, X.Y. Wang et al. XY22-947 (neotype: KUN, designated here; isoneotype: SDNU).

A more detailed description is in Magnusson (1929).

##### Note.

The type specimen was originally deposited at the Botanical Museum in Berlin-Dahlem (Magnusson 1929) but has unfortunately been lost during World War II (Dr. Bibiana Moncada, curator of the Herbarium of the Botanischer Garten Berlin (B), personal communication). According to the protologue, the original specimen was collected from Hami (historically referred to as “Chami”) in China. Based on the original description, ecology, chemistry, and specimens from Hami, we confirmed the taxonomic identity of this species and provided supporting molecular data. In addition to its occurrence in Xinjiang, this species is also distributed in Gansu, Qinghai, and Neimenggu provinces in China (Magnusson 1940, 1944).

**Figure 9.**
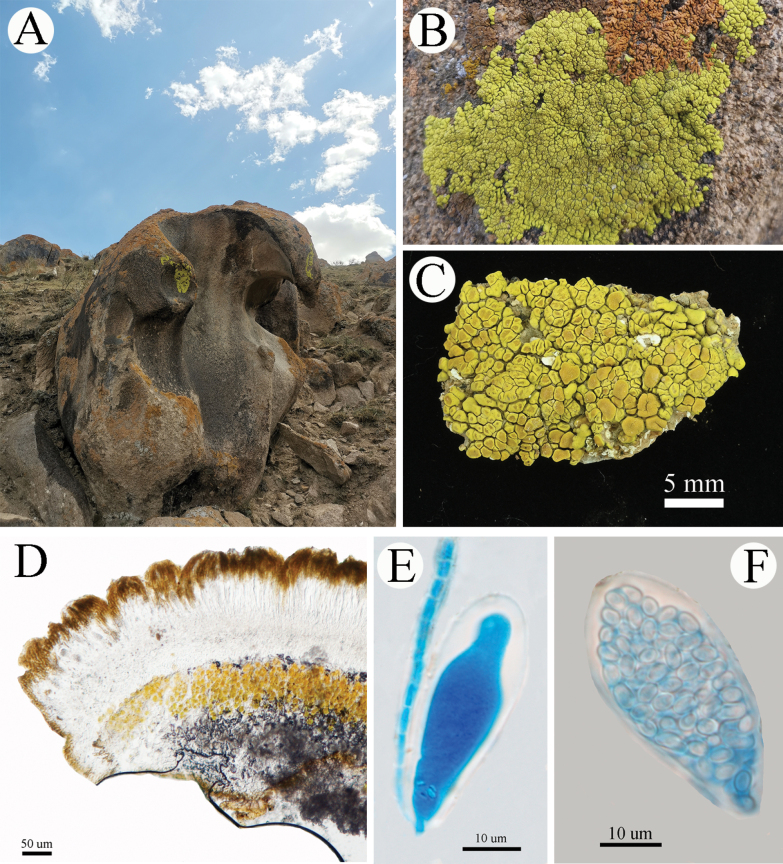
*Pleopsidiumgobiense* (neotype, XY22-947 KUN). A. General habitat; B. Thallus; C. Apothecia; D. Section of apothecium; E. Young ascus and paraphysis; F. Simple ascospores. E, F were stained with Lugol’s iodine solution. Scale bars: 10 µm (E, F); 50 µm (D); 5 mm (C).

##### Selected specimens examined.

China. • **Xinjiang Prov.**: Hami Ci., Balikun Co., G335, 43°36'10"N, 92°44'44"E, alt. 1634 m, 4 Jul. 2022, L.S. Wang et al. 22-72982 (KUN); • Hami Ci., Balikun Co., Haiziyan vil., 43°36'08"N, 92°44'42"E, alt. 1663 m, 4 Jul. 2022, D. Liu LD22-513 (KUN); • Zhaosu Co., Yuhu Lake, 81°03'03"N, 42°44'03"E, alt. 2010 m, 14 Sep. 2023, S.B. Zhang et al. XJ-ZSB23-119 (KUN).

### ﻿Key to the species of *Pleopsidium*

**Table:** 

1	Thallus with fatty acid(s)	**2**
–	Thallus without fatty acid(s)	**4**
2	Areoles elongated with distinctly radiating branches or not, thallus thin, distributed in alpine areas of Asia	***P.discurrens***
–	Without distinctly radiating elongated areoles, distributed in Europe and North America	**3**
3	Apothecia 1–3 mm in diam	***P.chlorophanum***
–	Apothecia less than 1 mm in diam	***P.flavum***
4	Thallus up to 3.0 mm thick, areoles convex and plump, lobes smooth and up to 4.5 mm long	***P.tumidulum***
–	Thallus less than 1.5 mm thick, areoles wrinkled, lobes less than 3 mm long	**5**
5	Apothecia frequent and plane, nearly flat to the thallus, pycnidia rare	***P.gobiense***
–	Apothecia rare, pycnidia frequent	***P.corrugatulum***

## Supplementary Material

XML Treatment for Pleopsidium corrugatulum

XML Treatment for Pleopsidium tumidulum

XML Treatment for Pleopsidium discurrens

XML Treatment for Pleopsidium gobiense

## Footnotes

For a detailed description, see Obermayer (1996).

## ﻿Additional information

### Use of AI

No use of AI was reported.

### Author ORCIDs

Min Ai https://orcid.org/0000-0002-7753-3310

Cong Cong Miao https://orcid.org/0009-0003-6450-8145

Fiona Ruth Worthy https://orcid.org/0000-0003-0042-3110

Xin Yu Wang https://orcid.org/0000-0003-2166-6111

### Data availability

All of the data that support the findings of this study are available in the main text or Supplementary Information.

## Supplementary materials

Supplementary material 1 The sequence of phylogenetic analysis: nuITS dataset Data type txt https://binary.pensoft.net/file/1393157This dataset is made available under the Open Database License (http://opendatacommons.org/licenses/odbl/1.0/). The Open Database License (ODbL) is a license agreement intended to allow users to freely share, modify, and use this Dataset while maintaining this same freedom for others, provided that the original source and author(s) are credited.Min Ai, Cong Cong Miao, Fiona Ruth Worthy, Lazzat Nurtai, De Ning Zhang, Sheng Bang Zhang, Gui Lan Han, Li Song Wang, Xin Yu Wang

Supplementary material 2 The sequence of phylogenetic analysis: nuLSU dataset Data type txt https://binary.pensoft.net/file/1393158This dataset is made available under the Open Database License (http://opendatacommons.org/licenses/odbl/1.0/). The Open Database License (ODbL) is a license agreement intended to allow users to freely share, modify, and use this Dataset while maintaining this same freedom for others, provided that the original source and author(s) are credited.Min Ai, Cong Cong Miao, Fiona Ruth Worthy, Lazzat Nurtai, De Ning Zhang, Sheng Bang Zhang, Gui Lan Han, Li Song Wang, Xin Yu Wang

Supplementary material 3 The sequence of phylogenetic analysis: the 3-locus concatenated matrix (nuITS-mtSSU-nuLSU) Data type txt https://binary.pensoft.net/file/1393159This dataset is made available under the Open Database License (http://opendatacommons.org/licenses/odbl/1.0/). The Open Database License (ODbL) is a license agreement intended to allow users to freely share, modify, and use this Dataset while maintaining this same freedom for others, provided that the original source and author(s) are credited.Min Ai, Cong Cong Miao, Fiona Ruth Worthy, Lazzat Nurtai, De Ning Zhang, Sheng Bang Zhang, Gui Lan Han, Li Song Wang, Xin Yu Wang

Supplementary material 4 BLASTn results for the nuLSU barcode sequences Data type docx Explanation note BLASTn results for the nuLSU barcode sequences designed for three species without fatty acids within *Pleopsidium*, under a scenario of corrected reference sequence labels (right annotation) based on phylogenetic inferences. Top-scoring hits are ranked by E-value and results with the lowest value are highlighted. https://binary.pensoft.net/file/1393160This dataset is made available under the Open Database License (http://opendatacommons.org/licenses/odbl/1.0/). The Open Database License (ODbL) is a license agreement intended to allow users to freely share, modify, and use this Dataset while maintaining this same freedom for others, provided that the original source and author(s) are credited.Min Ai, Cong Cong Miao, Fiona Ruth Worthy, Lazzat Nurtai, De Ning Zhang, Sheng Bang Zhang, Gui Lan Han, Li Song Wang, Xin Yu Wang
