# Supplementary material for: ﻿A new revision of the lichen genus Pleopsidium (Acarosporales, Acarosporaceae) in China reveals two new species
Source: MycoKeys. 2025 Aug 12;120:255–76. doi: 10.3897/mycokeys.120.161566 (PMC12365682; doi:10.3897/mycokeys.120.161566)
Supplement: Supplementary material 4 — BLASTn results for the nuLSU barcode sequences [file mycokeys-120-255-s004.docx]

**Figure 1.** BLASTn results for the nuLSU barcode sequences designed for three species without fatty acids within *Pleopsidium*, under a scenario of corrected reference sequence labels (right annotation) based on phylogenetic inferences. Top-scoring hits are ranked by E-value and results with the lowest value are highlighted.


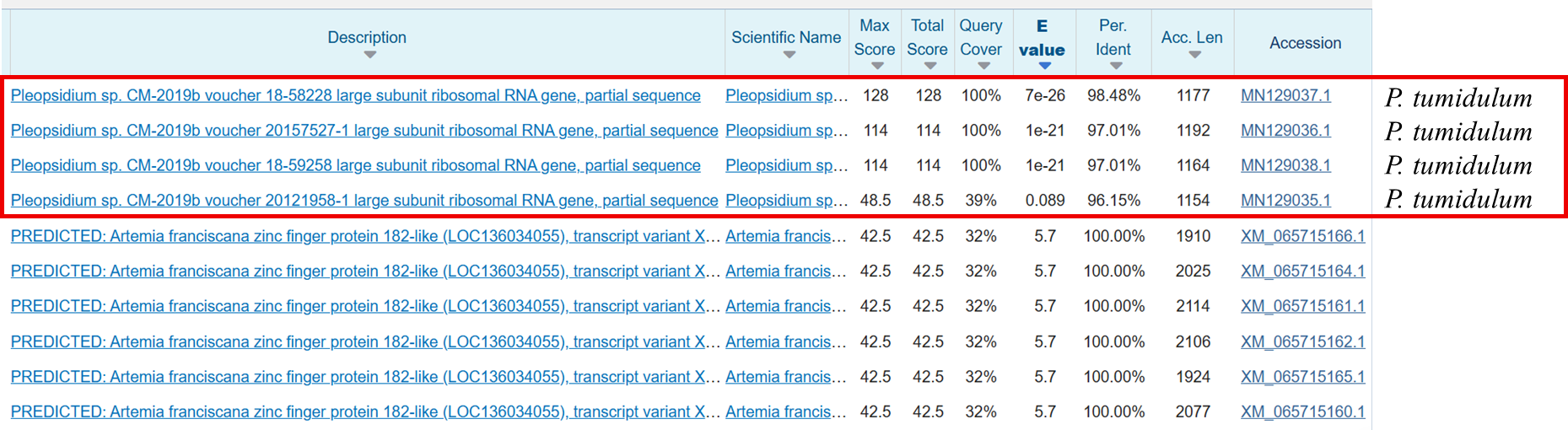


**A.** BLASTn top-scoring hits for the barcode sequence designed for *P. tumidulum*, nuLSU barcode:

TGTTGGCAGCACGTKAAAGATTGTCCAGTTTGTAGAGTATACATTCGAAAAGTCTTTCTCCGCAGC

Note: perfect identity scores (100%) could not be obtained due to the presence of ambiguous nucleotides in the queries.


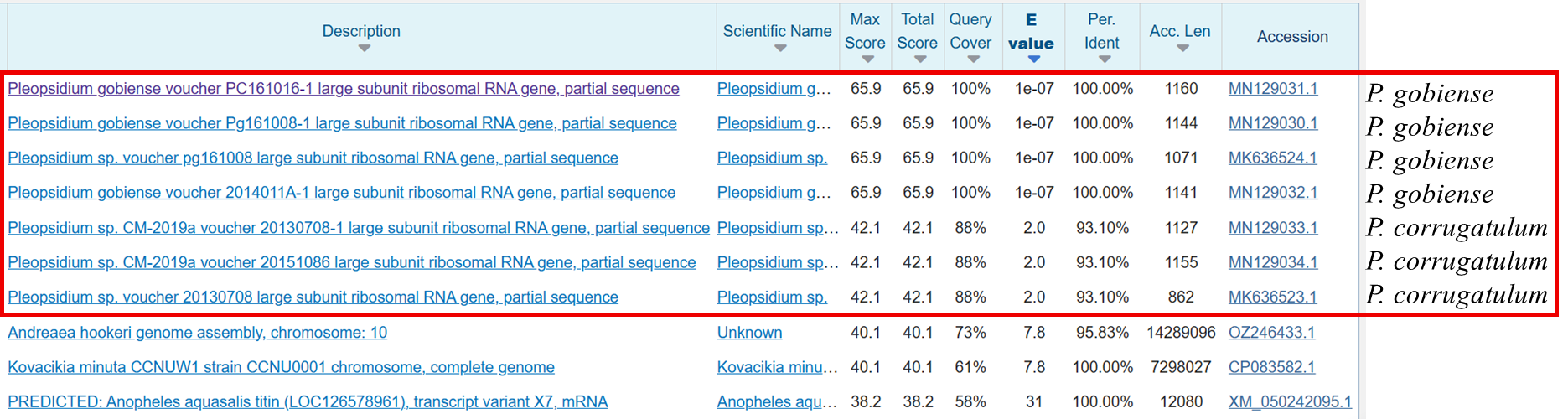


**B.** BLASTn top-scoring hits for the barcode sequence designed for *P. gobiense*, nuLSU barcode:

CATTGGCGGCACAGCAATGATTTGTCCCGTAGC


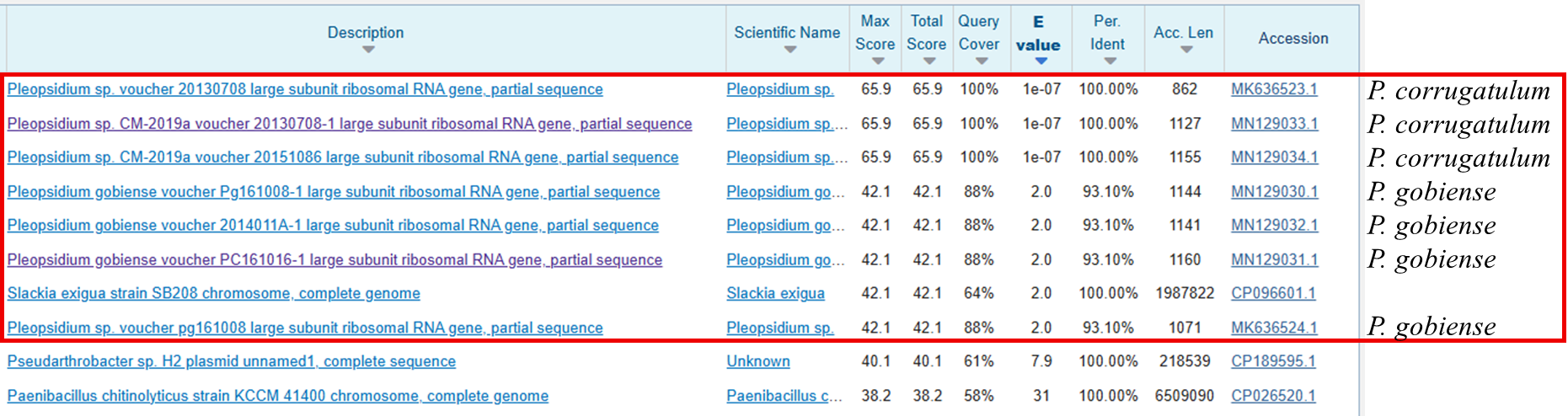


**C.** BLASTn top-scoring hits for the barcode sequence designed for *P. corrugatulum*, nuLSU barcode:

CATTGGCGGCACATGAATGATTTGTCCCGCGGC
